# AI-based quantification of whole-body tumour burden on somatostatin receptor PET/CT

**DOI:** 10.1186/s41824-023-00172-7

**Published:** 2023-08-07

**Authors:** Anni Gålne, Olof Enqvist, Anna Sundlöv, Kristian Valind, David Minarik, Elin Trägårdh

**Affiliations:** 1 Department of Medical Imaging and Physiology, Skåne University Hospital, Lund, Sweden; 2 Department of Translational Medicine, Faculty of Medicine, Lund University, Malmö, Sweden; 3 WCMM Wallenberg Centre for Molecular Medicine, Lund, Sweden; 4 Eigenvision AB, Malmö, Sweden; 5 Department of Electrical Engineering, Chalmers University of Technology, Gothenburg, Sweden; 6 Department of Clinical Sciences, Oncology and Pathology, Lund University, Lund, Sweden; 7 Radiation Physics, Skåne University Hospital, Malmö, Sweden; 8 Department of Medical Imaging and Physiology, Skåne University Hospital, Malmö, Sweden

**Keywords:** AI, Somatostatin receptor-expressing tumour volume, [^68^Ga]Ga-DOTA-TATE, [^68^Ga]Ga-DOTA-TOC, PET/CT

## Abstract

**Background:**

Segmenting the whole-body somatostatin receptor-expressing tumour volume (SRETVwb) on positron emission tomography/computed tomography (PET/CT) images is highly time-consuming but has shown value as an independent prognostic factor for survival. An automatic method to measure SRETVwb could improve disease status assessment and provide a tool for prognostication. This study aimed to develop an artificial intelligence (AI)-based method to detect and quantify SRETVwb and total lesion somatostatin receptor expression (TLSREwb) from [^68^Ga]Ga-DOTA-TOC/TATE PET/CT images.

**Methods:**

A UNet3D convolutional neural network (CNN) was used to train an AI model with [^68^Ga]Ga-DOTA-TOC/TATE PET/CT images, where all tumours were manually segmented with a semi-automatic method. The training set consisted of 148 patients, of which 108 had PET-positive tumours. The test group consisted of 30 patients, of which 25 had PET-positive tumours. Two physicians segmented tumours in the test group for comparison with the AI model.

**Results:**

There were good correlations between the segmented SRETVwb and TLSREwb by the AI model and the physicians, with Spearman rank correlation coefficients of *r* = 0.78 and *r* = 0.73, respectively, for SRETVwb and *r *= 0.83 and *r* = 0.81, respectively, for TLSREwb. The sensitivity on a lesion detection level was 80% and 79%, and the positive predictive value was 83% and 84% when comparing the AI model with the two physicians.

**Conclusion:**

It was possible to develop an AI model to segment SRETVwb and TLSREwb with high performance. A fully automated method makes quantification of tumour burden achievable and has the potential to be more widely used when assessing PET/CT images.

## Background

Neuroendocrine neoplasms (NENs) can be divided into often more indolent neuroendocrine tumours (NETs) and more aggressive neuroendocrine carcinomas (NECs) depending on morphology and proliferation rate (WHO 2019). Well-differentiated NETs typically overexpress somatostatin receptors (SSTR), which can be targeted in imaging and treatment (Bozkurt et al. 2017; Kaderli et al. 2019). An important imaging method for visualizing SSTR-expressing tumours is positron-emitting ^68^Ga-labelled somatostatin analogues such as [^68^Ga]Ga-DOTA-TOC, [^68^Ga]Ga-DOTA-TATE or [^68^Ga]Ga-DOTA-NOC during hybrid imaging with positron emission tomography and computed tomography (PET/CT) (Bozkurt et al. 2017).

Clinical interpretation of PET/CT images is a visually subjective assessment, sometimes with manual measurements of maximum standardized uptake value (SUVmax) in PET images and manual measurements of the largest tumour diameter in the CT images. Although the interpretation of SSTR PET/CT has shown good inter-observer agreement, differences between readers still exist and can be clinically significant, particularly when selecting patients for peptide receptor radionuclide therapy (PRRT) (Fendler et al. 2017). High tumour uptake correlates with well-differentiated tumours, longer progression-free survival (PFS), and improved response to PRRT (Campana et al. 2010; Kratochwil et al. 2015). However, SUVmax has several disadvantages, including its representation of only one voxel within the volume of interest (VOI), which means that the whole tumour burden is not represented, and its sensitivity to noise (Foster et al. 2014). Manual or semi-automatic drawing of volumetric measurements has numerous weaknesses, particularly time constraints and inter- and intra-observer variance (Foster et al. 2014). In the clinical setting, the evaluation of PET/CT images by nuclear medicine specialists and radiologists is challenging because of the time-intensive nature of the manual analysis, lack of quantification, and limitations in reproducibility regarding evaluation and quantification.

A method for measuring somatostatin receptor-expressing tumour volume (SRETV) and total lesion somatostatin receptor expression (TLSRE) on [^68^Ga]Ga-DOTA-TATE PET/CT has previously been suggested (Abdulrezzak et al. 2016). SRETV was measured as the tumour volume measuring more than 50% of SUVmax in a VOI, while TLSRE is calculated as the product of SRETV and SUVmean of each lesion (Abdulrezzak et al. 2016). Toriihara et al. evaluated the sum of whole-body SRETV (SRETVwb) and the sum of whole-body TLSRE (TLSREwb) concerning PFS. A larger tumour burden, measured as SRETVwb, showed significantly shorter PFS and may therefore have prognostic value (Toriihara et al. 2019). Similarly, other methods for measuring SRETVwb and TLSREwb have shown that higher tumour volume independently correlates with shorter PFS in both prospective and retrospective studies (Tirosh et al. 2017; Thuillier et al. 2022; Chen et al. 2023).

Deep learning is a subfield of machine learning, and artificial intelligence (AI) is a broader term. In the speciality of image analysis, the primary deep learning method chosen is convolutional neural networks (CNN) (Goodfellow and Courville 2016). CNNs are provided with large amounts of data, and the learning procedure imitates how information is processed through the brain, with some information processed through convolutions in numerous layers that activate different “neurons,” and in the end, there is an output, often as a set of classifiers, such as tumour lesion; yes or no (Alzubaidi et al. 2021).

A method to detect hepatic lesions on [^68^Ga]Ga-DOTA-TATE PET/CT with AI has been developed by Wehrend et al. (2021), and more recently, Carlsen et al. developed and implemented a CNN-based method to segment total tumour burden on [^64^Cu]Cu-DOTA-TATE PET/CT (Carlsen et al. 2022).

An objective automated method to detect and quantify SSTR-expressing tumour burden could improve the assessment of disease status and response evaluation, provide a tool for prognostication and potential prediction of treatment response, and improve the evaluation of response. Therefore, this study aimed to develop a method to detect and quantify SRETVwb and TLSREwb from [^68^Ga]Ga-DOTA-TOC and -TATE PET/CT images using CNNs.

## Methods

Adults (≥ 18 years) with a clinical indication for [^68^Ga]Ga-DOTA-TOC or -TATE PET/CT between August 2017 and December 2021, who were included in our larger study for validation of PET/CT, were eligible for this retrospective image analysis. Among these 848 patients, a subset of 200 patients were arbitrary selected for further analysis. The selection was made in a random manner without taking clinical or imaging data into account. Due to the time-intensive nature of segmenting tumours, including all patients was impossible. Exclusion criteria for further analysis in this study were inclusion in a future study planned to validate this method, incomplete examination, or larger extravasation of radiotracer outside the patient, such as urine. The study was carried out following the Declaration of Helsinki and was approved by the Swedish Ethical Review Authority (EPN LU 2016/417, 2018/753, and 2021-05734-02). All patients provided written informed consent. Data on age, sex, medical referral, and type of radiopharmaceutical were available for all patients. Clinical data on histopathological diagnosis, Ki-67, type of earlier or ongoing treatment, and TNM stage were verified by reviewing the patient’s digital medical record in the test group.

### PET/CT protocols

The PET/CT scans were performed at Skåne University Hospital in Lund using a Discovery MI or Discovery D690 (GE Healthcare, Milwaukee, USA) PET/CT system. In 2019, there was a shift in production from [^68^Ga]Ga-DOTA-TATE to [^68^Ga]Ga-DOTA-TOC, which is why both radiotracers were included in this study. The radiotracers were prepared according to established techniques (Bozkurt et al. 2017; Zhernosekov et al. 2007; Mueller et al. 2012). The patient received an intravenous injection of 2.0–2.5 MBq/kg activity (minimum administered activity 100 MBq and maximum 300 MBq), followed by a PET/CT scan approximately 60 min later. The scan covered from the base of the skull to mid-thigh. The PET acquisition time was 3.0–3 min 15 s per bed position, depending on the radiotracer and PET/CT system. Both systems used time-of-flight and point-spread function correction, and either a low-dose CT scan or a diagnostic CT with intravenous contrast (if there were no contraindications) was performed for attenuation correction and anatomic correlation.

### Manual image analyses

The images had previously been analysed by an experienced nuclear medicine physician and a radiologist in a clinical setting. For the current study, retrospective segmentation of tumours was performed in consensus by a PhD student in nuclear medicine/senior radiology resident (AG) and an experienced nuclear medicine physician (ET) using Hermes software (Hermes Medical Solutions, Stockholm, Sweden) and correlated with the clinical report. Uptake of [^68^Ga]Ga-DOTA-TOC and -TATE was considered significant for tumour segmentation if it did not correspond to physiological uptake. The PET/CT examination clinical report was used as a reference for segmentation. No predefined threshold for SUVmax was used. A semi-automatic method was used to delineate the tumours on the PET/CT images, where SRETV was defined as the tumour volume with uptake higher than 50% of SUVmax within a VOI, and TLSRE was defined as the product of SRETV and mean SUV (SUVmean) of the tumour volume (Abdulrezzak et al. 2016). Due to relatively high normal background uptake in the liver, manually drawn VOIs were often needed to avoid physiological uptake, as previously described (Toriihara et al. 2019). Overlap between tumour volumes was avoided. When a confluent uptake from closely related lesions was present, manually drawn VOIs around each lesion guided by the CT were needed to separate the lesions and avoid uptake from adjacent lesions. Inside the VOI, an irregular tumour volume with voxels higher than 50% of SUVmax was automatically obtained.

Whole-body tumour burden was defined as the sum of all SRETV and was described as SRETVwb. Whole-body somatostatin receptor expression was defined as the sum of all TLSRE and was described as TLSREwb. The semi-automatic tumour segmentations in the test group were separately performed by two physicians for comparison: Reader A (AG, ground truth) and Reader B (KV). Reader B was blinded to clinical parameters in correlation with the AI model.

### Training the AI model

At the core of our AI model was a UNet3D CNN that takes four types of input: a CT patch, a SUV patch, an organ mask patch (Trägårdh et al. 2020), and a SUV ratio patch with the ratio of the pixel SUV to the closest local maximum. The last patch was intended to enable thresholding at 50% of the lesion SUVmax (as used for the annotations). Meyer’s flooding algorithm on the SUV image determined the closest local maximum.

Before feeding them to the network, all inputs were resampled to 2.5 × 2.5 × 2.5 mm. CT patches were resampled using trilinear interpolation, but the nearest neighbour was used for SUV data and manual target segmentations, as the desired segmentation might depend on the exact relationship between SUV pixel values. The neural network output has the same voxel size, and to avoid introducing artefacts, this voxel size is maintained during post-processing and statistical analysis. The latter is first resampled to the CNN resolution for pixel-wise comparison to manual segmentations. Resampling the input patches also creates a consistent receptive field size for the network, regardless of the native image resolution. A UNet3D has a receptive field size of roughly 100 pixels (in any dimension), corresponding to around 25 cm in PET/CT images.

Before training of the AI model, the PET/CT examinations were divided into groups for training, validation, and testing. Random sampling was used for splitting the dataset into these groups. The number of patients in each group was set to 17% for testing and 17% for validation and the rest for training, according to the recommended ratio of dividing the training set in approximately 80% for training and 20% for validation (Courville IJGaYBaA. 2016). Usually the same number of data is used in the test group as in the validation group (Hastie et al. 2001). Training a neural network involves providing the network with a sequence of annotated image patches, and the way patches are chosen can affect the resulting network’s performance. Throughout the training, efforts are made to sample all images equally and preserve the balance between cancer and non-cancer images. Starting from uniform sampling within each class, the sampling of background voxels was updated in the same way as previously described in Trägårdh et al., while uniform sampling was maintained for the foreground (Trägårdh et al. 2022).

**Fig. 1:**
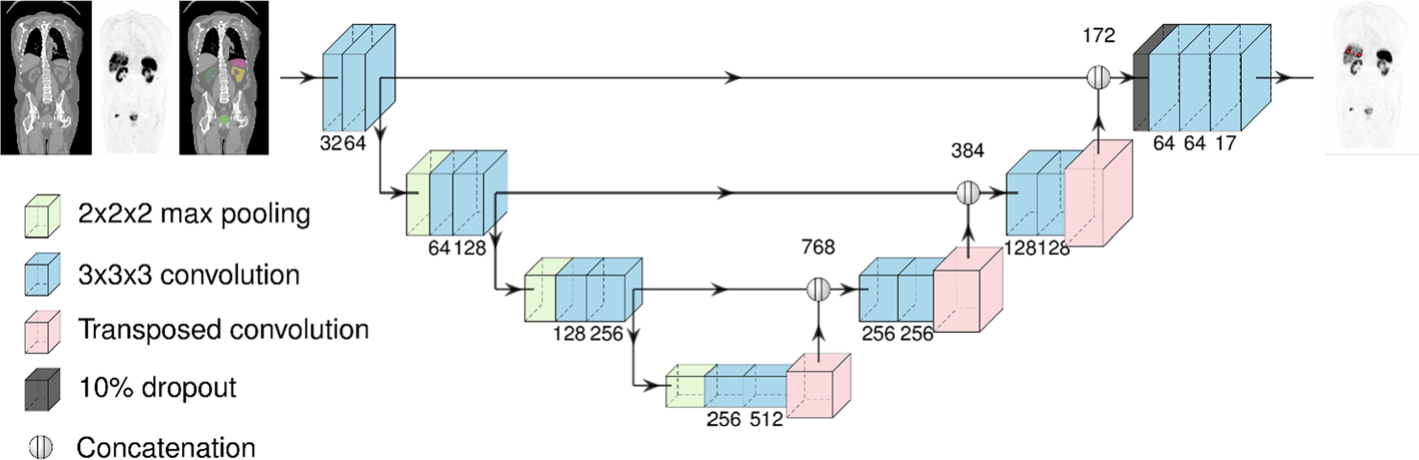
Illustration of the UNet3D architecture

For the actual optimization, the Adam optimizer with Nesterov momentum was used. The learning rate was initialized to 10^–4^ and reduced by a factor of 0.95 for every 20,000 samples until it reaches 10^–7^. To reduce overfitting, L2 regularization with a weight of 2 × 10^–4^ and a single dropout layer with a probability of 0.1 was used. The UNet3D architecture is illustrated in Fig. 1.

Connected components in the lesion mask were referred to as lesions. Post-processing was performed with the automatic removal of any lesions with a volume less than 0.05 ml, as it was found on the validation set that these were often false positives.

### Statistical analysis

A lesion having partial or full overlap with the reference segmentation was defined as a true positive lesion. A segmented lesion with no overlap with the reference segmentation was defined as a false positive lesion. Lastly, a lesion in the reference segmentation not segmented by the compared segmentation was defined as a false negative lesion. True negative lesions were excluded as it is impossible to define true negative lesions that were not detected. Sensitivity was calculated on a lesion detection level as the percentage of detected lesions compared to the reference segmentation. Positive predictive value (PPV) was calculated as the percentage of true positive lesions divided by the sum of true positive and false positive lesions, compared to the reference. Specificity could not be calculated since the AI model did not detect true negative lesions.

Calculations for true positives, false positives, false negatives, sensitivity, and PPV were made by comparing the AI model segmentations with both Reader A and Reader B, and Reader B with the ground truth Reader A.

The AI model’s measurements and the Readers’ measurements of SRETVwb and TLSREwb were calculated and compared. The correlation between the AI model’s measurements and the Readers’ measurements of SRETVwb and TLSREwb was assessed using Spearman rank correlation with a 2-tailed test, and it was considered statistically significant with a *p*-value < 0.05. The correlation was considered very strong for Spearman’s coefficient r > 0.8, moderately strong for values between 0.6 and 0.8, fair for values between 0.3 and 0.5, and poor for values below 0.3 (Chan 2003). Bland–Altman analysis was used to visually assess the level of agreement between the AI model and the manual SRETVwb and TLSREwb measurements. IBM SPSS Statistics version 28 was used for all statistical analyses.

## Results

### Study population

**Fig. 2:**
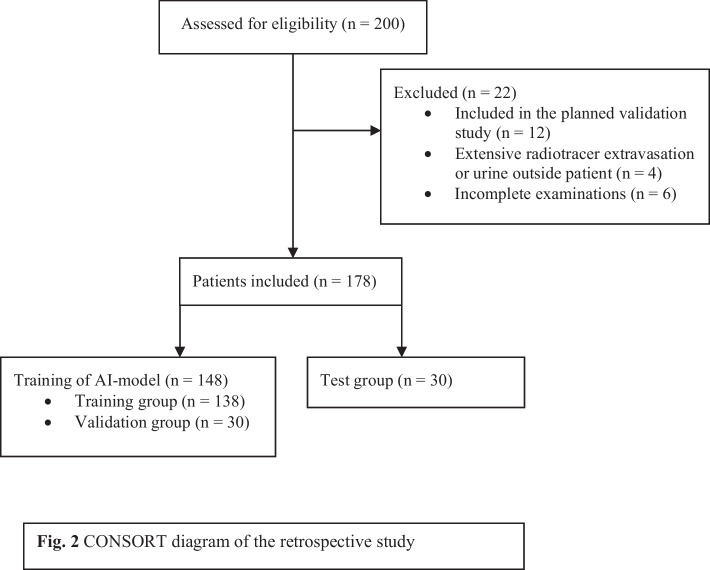
CONSORT diagram of the retrospective study

The study involved 200 PET/CT scans from 200 patients who underwent a clinically necessary [^68^Ga]Ga-DOTA-TOC or -TATE PET/CT at Skåne University Hospital between August 2017 and December 2021. Of these, 22 patients were excluded based on the exclusion criteria. The CONSORT diagram (Consolidated Standards of Reporting Trials) (Fig. 2) provides additional information. Ultimately, 178 patients were included and evaluated for SSTR-expressing tumour burden. The training and validation set comprised PET/CT scans from 148 patients, of whom 108 patients had suspected tumour lesions segmented, and 40 patients had no SSTR-expressing tumours. The radiotracer used in the training and validation set was [^68^Ga]Ga-DOTA-TATE in 44 examinations and [^68^Ga]Ga-DOTA-TOC in 104 examinations.

### Test group

The test group comprised 30 patients whose characteristics are presented in Table 1. Sixteen patients were female, and the mean age at the time of PET/CT was 66.4 years. Before the PET/CT, 23 patients had an established NEN diagnosis, and the mean age since diagnosis was 5.6 years (standard deviation, SD 4.0). Four patients were diagnosed after the PET/CT, bringing the total number of confirmed NEN diagnoses to 27. Of these, 15 patients had grade 1, five had grade 2 and one had grade 3. In six patients, the grade was unknown or not applicable.

**Table 1:** Patient characteristics in test group

Characteristic	Value (*n* = 30)
*Sex*	
Female	16
Mean age at PET/CT (y)	66.4 (SD 12.8)
*PET with*	
DOTA-TATE	9
DOTA-TOC	21
Low-dose CT	7
Diagnostic CT with iv. contrast	21
Diagnostic CT without iv. contrast	2
*Reason for PET/CT*	
First examination	7
Follow-up after treatment	13
Follow-up after surgery	1
Suspected progressive disease	5
Evaluation before decision about PRRT	4
*Primary tumour*	
No verified NET and no SSTR-expressing lesion	3
Small bowel	18
Pancreas	3
Medullary thyroid cancer	2
Stomach	1
MEN 1	1
Atypical carcinoid	1
Small cell neuroendocrine carcinoma	1
*Ki-67 (%)*	
< 3	17
3 to ≤ 20	7
> 20	2
Unknown	1
*Earlier treatment before PET/CT*	
Surgery	22
PRRT	2
Chemotherapy	2
Somatostatin analog	1
Ongoing treatment with somatostatin analog	13
*TNM stage at PET/CT*	
Only primary tumour	5
Loco-regional disease	2
Metastatic	18
NET verified pathological diagnose but no remaining tumour on PET/CT	2
No NET	3
Distribution of somatostatin receptor-expressing lesions*	
Pathological uptake	25
Liver	7
Lung	3
Bone	9
Abdomen (except liver)	9
Lymph nodes	10
Other locations	4
No pathological uptake	5

*The total number of the distribution of somatostatin receptor-expressing lesions is more than 25 as patients could have uptake on several locations

### Performance of the AI model

Out of the 27 patients diagnosed with NEN, 25 had SSTR-expressing tumour lesions, and the AI model classified 24 of these patients as positive and 3 out of 5 patients as negative. When Reader A was used as a reference, Reader B classified 24 out of 25 patients as true positive and 5 as true negative. Details on image classification at the patient level are summarized in Table 2. Reader A (ground truth) segmented 267 lesions on a lesion detection level, with a median of 3 lesions per patient and a range of 0–58 lesions. The median segmented tumour volume SRETVwb was 6.8 ml for Reader A, 4.7 ml for the AI model, and 6.5 ml for Reader B. Additional details on segmented lesions and tumour volume are displayed in Table 3. The sensitivity on a lesion detection level was 80% for the AI model versus Reader A and 79% versus Reader B. When comparing Reader B versus Reader A, the sensitivity was 92%. Details on sensitivity and PPV are presented in Table 4.

**Table 2:** Classification of images on patient level

	AI model versus Reader A	AI model versus Reader B	Reader B versus Reader A
Correct classification of PET-CT image	27	26	29
True positive patient	24	23	24
True negative patient	3	3	5
False positive patient	2	3	0
False negative patient	1	1	1

True positive, true negative, false positive and false negative classification of images on patient level when using manual segmentation or reference doctor as comparison. The results are presented for the entire test group (*n* = 30)

**Table 3:** Segmented lesions in the test group

	Reader A	AI model	Reader B
Total number of segmented lesions	267	265	269
Number of lesions per patient (IQR)	3.0 (1.0–14.8)	3.5 (1.75–14.8)	2.5 (1.0–16.3)
SRETVwb (IQR)	6.8 (1.0–35.8)	4.7 (0.5–28.8)	6.5 (0.5–42.8)
TLSREwb (IQR)	61.9 (7.6–583.4)	40.7 (3.4–562.1)	65.1 (2.1–605.0)

Total number of segmented lesions in the test group (*n* = 30) presented for Reader A, the AI model and reference Reader B. Numbers are total number of lesions, median number of lesions per patient, whole-body somatostatin receptor-expressing tumour volume (SRETVwb) in ml and whole-body total lesion somatostatin receptor expression TLSREwb, with interquartile range (IQR)

**Table 4:** Classification of segmented lesions

	AI model versus Reader A	AI model versus Reader B	Reader B versus Reader A
*True positive lesions*			
Total	214	210	246
Per patient (IQR)	2 (0.0–12.0)	1 (0.0–12.0)	2.5 (1.0–14.5)
*False positive lesions*			
Total	45	41	25
Per patient (IQR)	1 (0.0–2.3)	1 (0.0–2.0)	0.0 (0.0–1.3)
*False negative lesions*			
Total	53	59	21
Per patient (IQR)	1 (0.0–2.0)	1 (0.0–3.3)	0.0 (0.0–1.0)
Sensitivity (%)	80	79	92
PPV (%)	83	84	91

Number of lesions which are true positive, false positive and false negative for the AI model versus Reader A, the AI model versus Reader B and Reader B versus Reader A. Values are total number of lesions, median number of lesions per patient, median volume (ml) and median TLSREwb with interquartile range (IQR). Sensitivity and positive predictive value (PPV) are also presented for each comparison

**Fig. 3:**
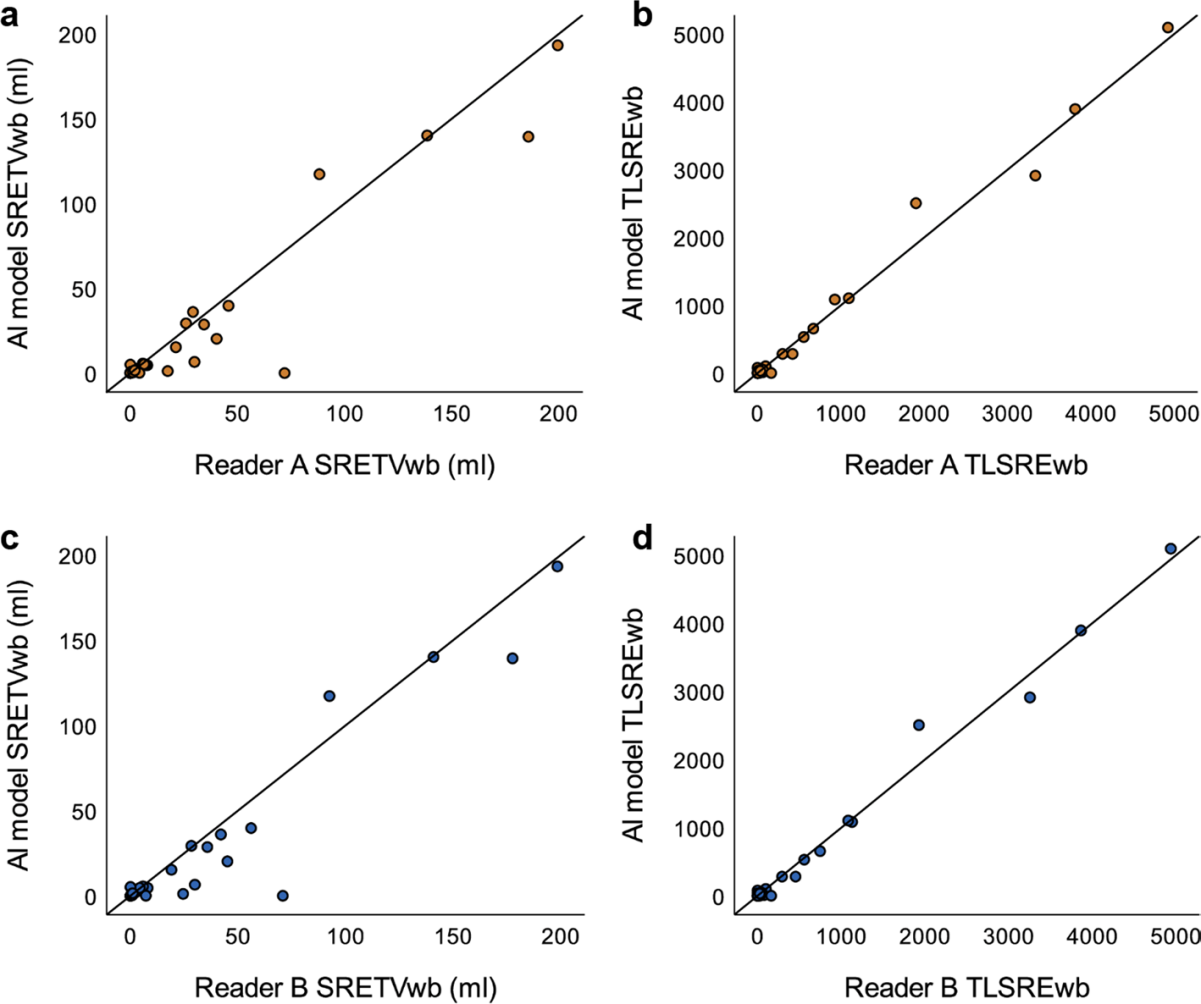
Scatter plots illustrating the correlation between the AI model and Reader A for SRETVwb, (*r* = 0.78, *p* < 0.001) (**a**), respectively, TLSREwb (*r* = 0.83, *p* < 0.001) (**b**) and between the AI model and Reader B for SRETVwb (*r* = 0.73, *p* < 0.001) (**c**), respectively, TLSREwb (*r* = 0.81, *p* < 0.001) (**d**)

**Fig. 4:**
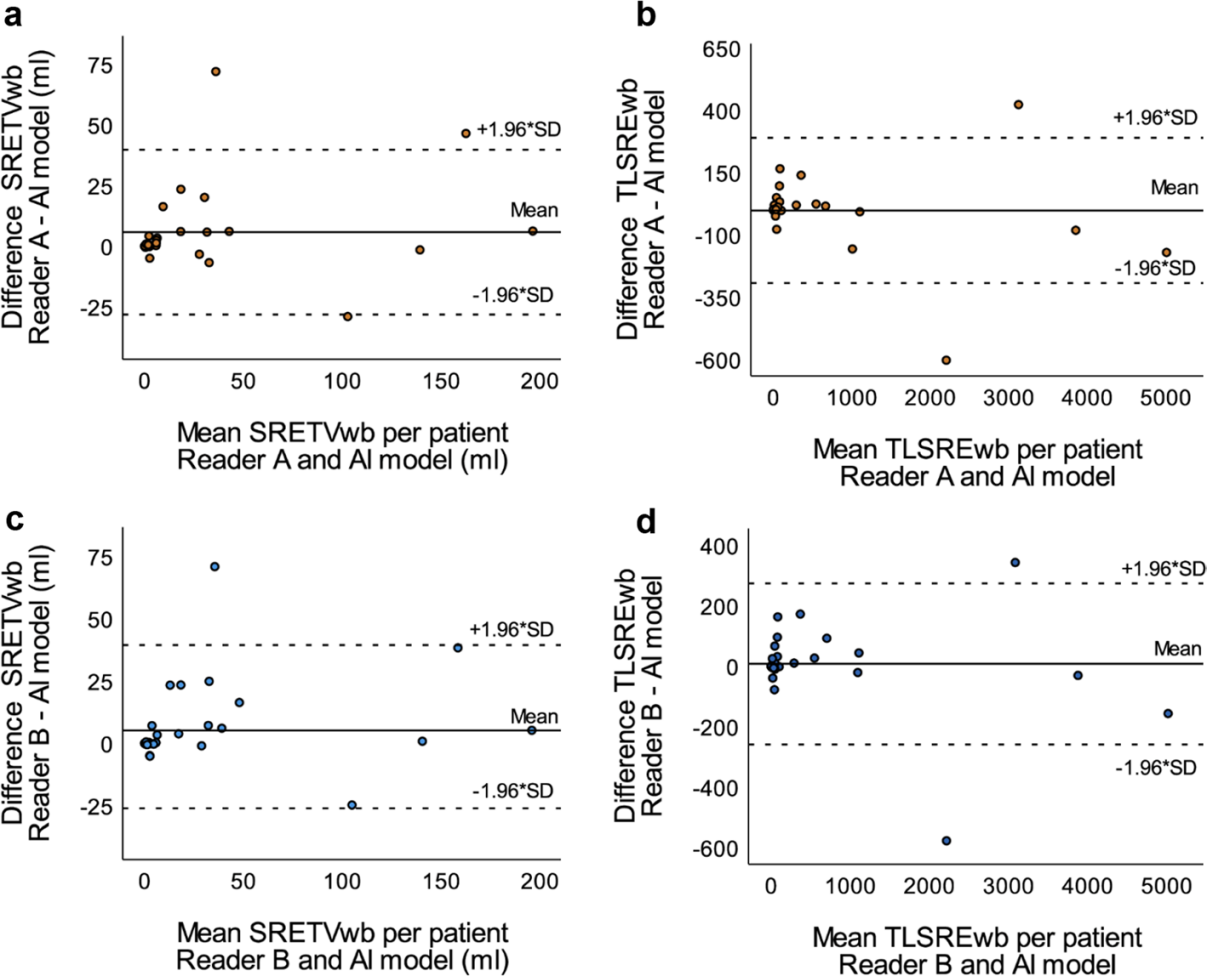
Bland–Altman plots illustrating the agreement of SRETVwb between the AI model and Reader A (**a**) and Reader B (**c**), respectively, as well as the agreement of TLSREwb between the AI model and Reader A (**b**) and Reader B (**d**). Dotted lines specify the 95% upper and lower limits of agreement

There was a moderately strong correlation (*r* = 0.78, *p* < 0.001) between SRETVwb for the AI model versus Reader A (Fig. 3a) and a very strong correlation (*r* = 0.83, *p* < 0.001) for TLSREwb (Fig. 3b). The values were similar for the AI model versus Reader B for both SRETVwb (Fig. 3c) and TLSREwb (Fig. 3d). The correlation between Reader B versus Reader A was even higher, with *r* = 0.96 (*p* < 0.001) for SRETVwb and *r* = 0.99 (*p* < 0.001) for TLSREwb. To demonstrate the agreement between the AI model versus Reader A and the AI model versus Reader B, Bland–Altman plots were created (Fig. 4).

**Fig. 5:**
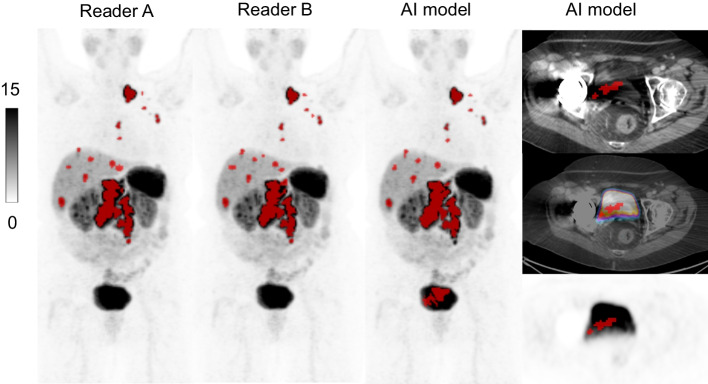
MIP-images with tumour segmentation for Reader A, Reader B and the AI model, respectively. To the right axial images over bladder with the AI model segmentations. A part of the bladder was included as tumour volume by the AI model. A unilateral hip prosthesis caused metal artefacts over the pelvic area, which might have contributed to this error. SRETVwb was 88.2 ml for Reader A, 92.5 ml for Reader B and 117.2 ml for the AI model

**Fig. 6:**
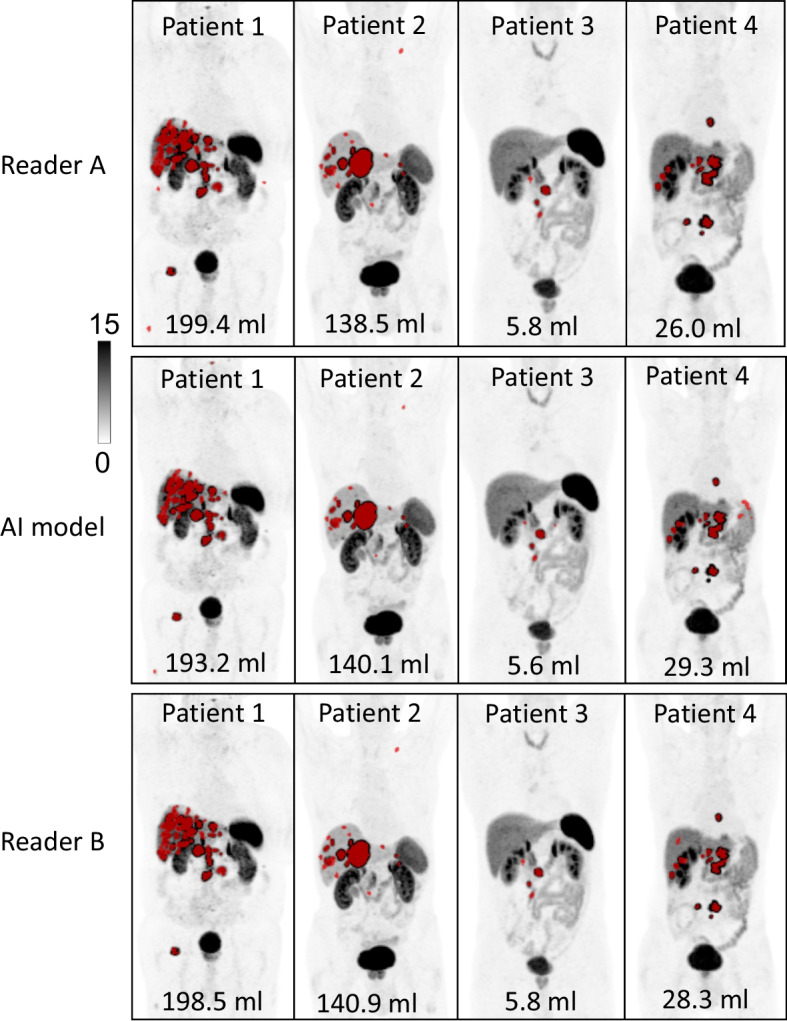
Demonstration of 4 patients were the AI model segmented tumours similar as Reader A and Reader B. SRETVwb are presented for each example

The AI model was less accurate in two patients, in which it segmented larger volumes of healthy organs, including part of the kidney in one patient and part of the bladder in the other. The likely reason for these mistakes was the presence of large benign cysts in the kidney of one patient, resulting in an unusual organ shape. In the other patient, a unilateral hip prosthesis caused metal artefacts over the bladder, making it difficult to distinguish on the CT images. An example of the error in the segmented bladder is illustrated in Fig. 5. Additionally, the AI model had difficulty segmenting mediastinal tumours, which are rarer than abdominal tumours and, therefore, less frequent in the training data. Similarly, tumours with very low uptake were challenging for the AI model to segment. One patient classified as negative by Reader A and B and positive by the AI model had only a tumour volume of 0.09 ml segmented by the AI model. These pixels were located in the spleen, but the CT images had significant breathing artefacts, which might have affected this segmentation. Overall, the segmentations by the AI model were consistent with those of Reader A and B, with some of the best examples of tumour segmentations demonstrated in Fig. 6.

## Discussion

Segmenting the whole-body tumour burden is time-consuming but has shown value in predicting PFS (Toriihara et al. 2019; Thuillier et al. 2022). It could be an important predictive factor when selecting patients for PRRT, individualizing PRRT, for prognostication and during follow-up (Ebbers et al. 2021). This is the first study to develop a completely automatic AI model to measure the whole-body somatostatin receptor-expressing tumour volume and whole-body total lesion somatostatin receptor expression (SRETVwb and TLSREwb) on [^68^Ga]Ga-DOTA-TOC and -TATE PET/CT images. Carlsen et al. recently developed a similar method for [^64^Cu]Cu-DOTA-TATE PET/CT, showing a similar lesion-wise sensitivity of 84.4% compared to 80% in our model. However, they excluded negative control patients from the test group, which makes the results not entirely comparable (Carlsen et al. 2022).

Our AI model’s measurements of SRETVwb and TLSREwb in the test group correlated strongly to very strongly with the segmentations made by Reader A and B. The AI model made a few obvious errors, but in those cases, reasonable explanations could be found, such as metal artefacts, large breathing artefacts, multiple cysts making the anatomy very different, or very low uptake in tumours. A large amount of training data is crucial, and a larger training set might improve these errors. Still, as this disease is quite rare (Dasari et al. 2017), it is also a limiting factor for training the AI model. As all patients are individuals, there could always be specific factors that an AI model has not encountered before, and therefore, it seems unlikely that AI tools will be able to stand alone in the near future. Approval of the segmentations by a physician is needed when implementing an AI model, as described by Carlsen et al. (2022). Additionally, post-processing using a higher cut-off volume than 0.05 ml for removal of larger lesions, such as 0.1 ml, as compared by Carlsen et al. (2022), might be of interest, which could have made the number of false positives lower. However, there is always a risk of leaving out too much information.

The majority of neuroendocrine tumours overexpresses the SSTR subtype 2 most, but the subtype receptor expression is associated with the type of tumour and differentiation (Reubi 2003). Both [^68^Ga]Ga-DOTA-TOC and -TATE share the same imaging characteristics (Velikyan et al. 2014) although their binding affinity for the subtypes of SSTR is slightly different (Reubi et al. 2000). When comparing the two tracers, the differences have been found to be small (Poeppel et al. 2011). Including both tracers might comprise a larger imaging diversity when training the AI model and we believe it is more of a strength than a weakness that both radiopharmaceuticals were included.

Random sampling was used for randomisation of the patients into training, validation and test groups. This method was used to minimize the risk of introducing bias to the model. A potential weakness is that the clinical parameters in the different groups may not have been evenly distributed.

Another limiting factor of this study is that manual segmentation is very time-consuming, and a semi-automatic method was used for segmenting all tumours. This method is imperfect, and like all segmentation methods, it has weaknesses (Foster et al. 2014; Im et al. 2018). We used this semi-automatic method of delineating 50% of SUVmax because it was previously described and showed value in predicting PFS (Abdulrezzak et al. 2016; Toriihara et al. 2019). It was also a method that could be implemented in our software and was feasible compared to complete manual segmentations, especially considering the high number of metastases that occurred in some of the patients.

Detectability of tumour lesions in the liver is complicated by a high normal background uptake. Normal hepatic uptake is also influenced by other factors, such as treatment with long-acting somatostatin analogues (Gålne et al. 2019) and the tumour “sink effect” (Beauregard et al. 2012), which likely affect the detection rate of liver metastases, both for manual detection and with AI models. One difference in our study compared to other studies exploring tumour burden is that we have segmented suspected tumour lesions outside the liver with lower uptake than the liver (Thuillier et al. 2022; Wehrend et al. 2021; Carlsen et al. 2022). We included these lesions in the model because tumour burden with an uptake lower than the liver could be important in the clinical setting. Lesions with the lowest SUVmean have shown increased prognostic value compared to the highest SUVmax in [^64^Cu]Cu-DOTA-TATE PET/CT (Carlsen et al. 2021). These lesions could be of a more aggressive nature with potential uptake of 2-[^18^F]fluoro-2-deoxy-D-glucose (2-[^18^F]FDG) if dual-imaging was performed (Chan et al. 2017) or could include necrosis after treatment with PRRT, thus showing treatment response. Segmentation of only lesions with higher uptake than the liver might provide a biased AI model, which would likely segment fewer false positive lesions, giving a higher PPV, potentially resulting in a model that would miss lesions of importance.

Inter-observer variability for SSTR PET/CT has shown consistent and good agreement, but still, both false positive and false negative interpretations of the overall scan result occur, as indicated in our results (Fendler et al. 2017). Some inter-observer variability is also expected because there is a difference in background information given to Reader A relative to the AI model and Reader B, who did not have access to the referral text or written clinical report.

A weakness of this study is the lack of external validation, as recently noted by Pantelis et al. in a review of AI and GEP-NEN (Pantelis et al. 2022). Although external validation was not in the scope of this study, it would have been a significant strength. For clinical validation of the method, we have planned another study to explore the value of the AI model measurements of SRETVwb and TLSREwb on [^68^Ga]Ga-DOTA-TOC and -TATE PET/CT images concerning PFS and OS after treatment with PRRT.

We do not believe that AI will make radiologists or nuclear medicine specialists obsolete in the near future, but rather increase the value of PET/CT examinations by providing validated imaging biomarkers, such as tumour volume. A future AI method will still require the physician to visually inspect the segmented tumour volume and correct obviously inaccurate lesions. We believe that automated AI models, such as in the field of imaging, could help to improve patient care with better prognostication, prediction of treatment response and for evaluation of follow-up examinations.

## Conclusion

To the best of our knowledge, this is the first fully automatic AI model to segment the whole-body somatostatin-expressing tumour volume, SRETVwb, and total lesion somatostatin receptor expression, TLSREwb, on [^68^Ga]Ga-DOTA-TOC and -TATE PET/CT images. Our AI model showed a good correlation with ground truth with few false positive and false negative lesions per patient. Since segmenting the tumour burden is a time-intensive task, it is not feasible in clinical practice. A clinically validated AI model segmenting neuroendocrine tumour lesions could be of prognostic, as well as predictive value when selecting patients for treatment.

## Data Availability

The datasets generated during the current study are not publicly available due to ethical considerations. Access to individual data can only be obtained after ethical approval from the Swedish Ethical Review Authority. The AI model is available upon reasonable request.

## Abbreviations

2-[^18^F]FDG: 2-[^18^F]fluoro-2-deoxy-d-glucose
AI: Artificial intelligence
CONSORT: Consolidated standards of reporting trials
CNN: Convolutional neural networks
CT: Computed tomography
SUVmax: Maximum standardized uptake value
SUVmean: Mean standardized uptake value
NEC: Neuroendocrine carcinoma
NEN: Neuroendocrine neoplasm
NET: Neuroendocrine tumour
PRRT: Peptide receptor radionuclide therapy
PPV: Positive predictive value
PET: Positron emission tomography
PFS: Progression-free survival
SSTR: Somatostatin receptor
SRETV: Somatostatin receptor-expressing tumour volume
SD: Standard deviation
TLSRE: Total lesion somatostatin receptor expression
VOI: Volume of interest
SRETVwb: Whole-body somatostatin receptor-expressing tumour volume
TLSREwb: Whole-body total lesion somatostatin receptor expression
